# The effect of supplemental oxygen on perioperative brain natriuretic peptide concentration in cardiac risk patients – a protocol for a prosprective randomized clinical trial

**DOI:** 10.1186/s13063-020-04336-9

**Published:** 2020-05-12

**Authors:** Christian Reiterer, Barbara Kabon, Markus Falkner von Sonnenburg, Patrick Starlinger, Alexander Taschner, Oliver Zotti, Julius Goshin, Gregor Drlicek, Edith Fleischmann

**Affiliations:** 1 Department of Anaesthesia, General Intensive Care Medicine and Pain Medicine, Medical University of Vienna, Spitalgasse 23, 1090 Vienna, Austria; 2 Department of Surgery, Medical University of Vienna, 1090 Vienna, Austria; 3 Franziskus Spital, Anaesthesia and Intensive Care, 1050 Vienna, Austria

## Abstract

**Background:**

Elevated postoperative *N*-terminal pro-B-type natriuretic peptide (NT-proBNP) concentrations are predictive for cardiac adverse events in noncardiac surgery. Studies indicate that supplemental oxygen decreases sympathetic nerve activity and might, therefore, improve cardiovascular function. Thus, we will test the effect of perioperative supplemental oxygen administration on NT-proBNP release after surgery.

**Methods/design:**

We will conduct a single-center, double-blinded, randomized trial at the Medical University of Vienna, including 260 patients with increased cardiac risk factors undergoing moderate- to high-risk noncardiac surgery. Patients will be randomly assigned to receive 80% versus 30% oxygen during surgery and for 2 h postoperatively. The primary outcome will be the difference in maximum NT-proBNP release after surgery.

As secondary outcomes we will assess the effect of supplemental oxygen on postoperative maximum troponin T concentration, oxidation-reduction potential, von Willebrand factor concentration and perioperative fluid requirements. We will perform outcome measurements 2 h after surgery, on postoperative day 1 and on postoperative day 3. The NT-proBNP concentration and the oxidation-reduction potential will also be measured within 72 h before discharge.

**Discussion:**

Our trial should determine whether perioperative supplemental oxygen administration will reduce the postoperative release of NT-proBNP in patients with preoperative increased cardiovascular risk factors undergoing noncardiac surgery.

**Trial registration:**

ClinicalTrials.gov, ID: NCT03366857. Registered on 8th December 2017.

## Background

Major cardiovascular complications occur in about 3% of all patients undergoing noncardiac surgery [1, 2]. In patients over the age of 45 years the risk of developing postoperative complications is even higher [2].

As supplemental oxygen has no effect on the incidence of surgical site infections, it is reasonable to question the effect of hyperoxia on other organ systems [3–6]. The systemic effect of perioperative supplemental oxygen, especially on the cardiovascular system, still remains unknown. A post-hoc analysis of the PROXI trial indicated an increased long-term risk of myocardial infarction or other heart diseases in patients receiving supplemental oxygen [7]. Despite the current controversy regarding toxic effects of supplemental oxygen [8], there are also studies indicating the beneficial effects of higher oxygen concentrations specifically on the cardiovascular system. A decrease in heart rate and myocardial oxygen consumption as well as an increase in myocardial oxygen supply was shown previously [9–11].

For example, nocturnal oxygen therapy significantly reduced the brain natriuretic peptide (BNP) concentration and prevented the progression of congestive heart failure in patients with central sleep apnea [12].

*N*-terminal pro-B-type natriuretic peptide (NT-proBNP) is a hormone, which is released during increased myocardial stress [13]. Measurement of NT-proBNP is recommended to enhance perioperative cardiac risk estimation in patients aged over 65 years of age or older, and also those aged 45–64 years of age with significant cardiovascular disease undergoing noncardiac surgery [14].

The preoperative assessment of NT-proBNP is recommended for patients at high risk of cardiovascular events undergoing noncardiac surgery [15]. Postoperatively elevated NT-proBNP concentrations are associated with increased postoperative cardiac mortality and cardiac failure within 30 days after noncardiac surgery [16].

No data are available regarding the effect of supplemental oxygen on postoperative NT-proBNP concentrations. Thus, we will test our hypothesis that perioperative supplemental oxygen (80% versus 30%) decreases the postoperative maximum NT-proBNP concentration assessed within 2 h, on postoperative day (POD) 1 and POD 3 in patients with increased cardiovascular risk factors undergoing moderate- to high-risk noncardiac surgery. Furthermore, we will evaluate differences of NT-proBNP concentration within 72 h before discharge between both study groups. Our secondary outcomes will test the effect of supplemental oxygen on postoperative troponin T (TnT) concentration, oxidation-reduction potential and von Willebrand factor (vWF) concentration and perioperative fluid requirements.

## Methods/design: data collection, management and analysis

### Objectives and design

We will conduct a single-center, double-blinded, two-arm trial at the Medical University of Vienna. In total, we will randomly assign 260 patients to receive 80% versus 30% inspired oxygen concentration for surgery and for 2 h postoperatively. We will follow the Standard Protocol Items: Recommendations for Interventional Trials (SPIRIT) recommendations for interventional trials (Additional file 1). The study was approved by the Ethics Committee of the Medical University of Vienna on 13 November 2017. This trial was registered at ClinicalTrials.gov on 8 December 2017 (NCT03388957) and at the European Clinical Trial Database (2017–003714-68).

This trial will test the primary hypothesis that supplemental oxygen significantly decreases the maximal postoperative NT-proBNP concentration (assessed within 2 h after surgery, on POD 1 and POD 3) in patients with increased cardiovascular risk factors undergoing moderate- to high-risk abdominal surgery.

Our secondary outcomes are differences in maximum TnT concentration, static oxidation-reduction potential (sORP) and oxidation-reduction potential capacity (cORP), as well as vWF values and the perioperative fluid requirements to reach hemodynamic stability (Table 1).

**Table 1:** Outcomes assessment

	Outcomes	Measurements
Primary	Myocardial function	maxNT-proBNP
Secondary	MINS	maxTnT
	Surgical stress	Copeptin
	Redox stress	sORP, cORP
	Hemodynamic	
Exploratory	Cardiac failure	50% increase in NT-proBNP
	Myocardial infarction	ECG change and elevated heart-specific enzymes
	Heart failure requiring intervention	50% NT-proBNP increase and medical treatment
	New onset of cardiac arrhythmias	ECG changes requiring medical treatment and/or electric cardioversion
	Unplanned ICU admission	

c*ORP* oxidation-reduction capacity, *ECG* electrocardiogram, *ICU* intensive care unit, *maxNT-proBNP* maximal *N*-terminal pro-B-type natriuretic peptide, *maxTnT* maximal troponin T, *MINS* Myocardial Injury after Noncardiac Surgery, *sORP* static oxidation-reduction potential

### Study population

**Fig. 1:**
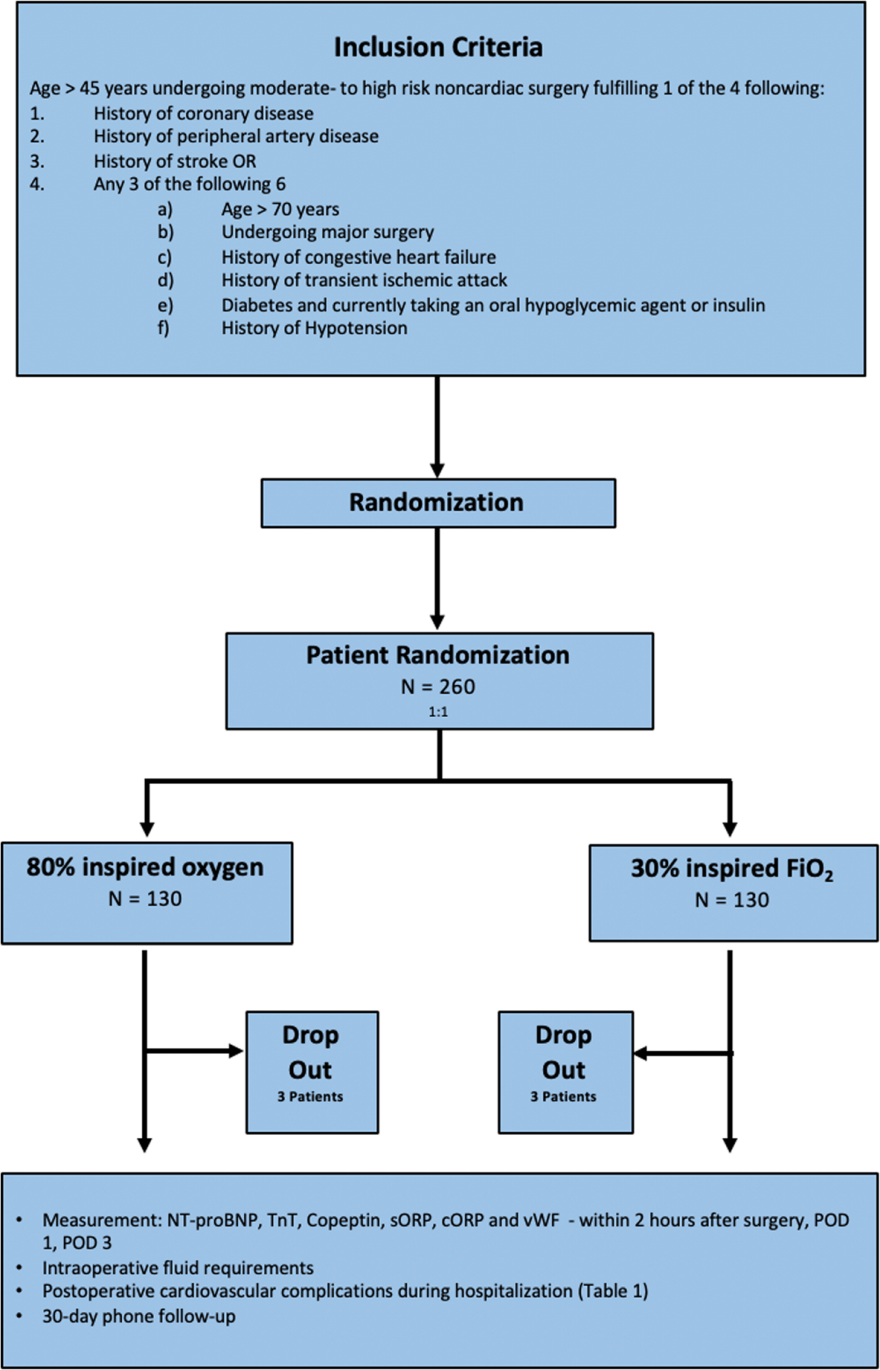
Study flow chart. *BNP* brain natriuretic peptide, *TnT* troponin T, *sORP* static oxidation-reduction potential, *cORP* oxidation-reduction potential capacity, *POD* postoperative day

We will screen for patients of at least 45 years of age planned for elective moderate- to high-risk abdominal surgery under general anesthesia with an expected duration of surgery of at least 2 h. Patients are eligible if they meet one or more the following criteria: (1) history of coronary artery disease; (2) history of peripheral arterial disease; (3) history of stroke o*r* (4) any three of these six criteria: (a) age over 70 years, (b) undergoing major surgery, (c) history of congestive heart failure, (d) history of transient ischemic attack, (e) diabetes and currently taking an orally administered hypoglycemic agent or insulin and (f) history of hypertension. Patients will be not eligible if they meet one of the following criteria: (1) symptoms of infection or sepsis; (2) preoperative inotropic therapy; (3) patients under intensive care unit (ICU) treatment; (4) oxygen-dependent patients and (5) history of severe heart failure and/or an ejection fraction < 30%. (Fig. 1).

### Protocol

We will perform standardized monitoring including electrocardiogram (ECG), blood pressure and oxygen saturation (SpO_2_) in all patients after arriving in the operating theater. Shortly before induction of anesthesia, patients will be randomly assigned to one of the two oxygen concentrations. We will administer 100% oxygen in all patients for induction of anesthesia according to clinical standards. We will induce anesthesia using 1–3 μg kg^− 1^ body weight (BW) fentanyl, 2 mg kg^− 1^ (BW) propofol and 0.6 mg kg^− 1^ rocuronium. In addition to standard monitoring, an arterial line will be inserted for direct blood pressure monitoring. A central venous line will be inserted at the discretion of the attending anesthesiologist.

Anesthesia will be maintained with sevoflurane (up to 1.5 minimum alveolar concentration (MAC)) in an oxygen gas carrier according to processed electroencephalogram (EEG)-guided anesthesia. Muscle relaxation will be given when necessary to maintain one to two mechanical twitches in response to supra-maximal stimulation (Train-of-Four stimulation, target < 75%). Temperature will be held at a core temperature over 36 °C using forced-air warming.

We will use esophageal-Doppler-guided (Cardiac Q, Deltex Medical, Chichester, UK) fluid management according to a previously published algorithm [17, 18]. All patients will receive a 2 mL/kg BW baseline infusion of balanced crystalloids. A bolus of 250 mL balanced crystalloids will be administered when the stroke volume decreases by more than 20% as compared to baseline values. In the case of acute bleeding or a systemic inflammatory response during surgery, volume will be administered according to fluid requirements to maintain hemodynamic stability. Blood and blood products will be administered per clinical judgment.

There will be no restrictions or prohibitions of medical care and therapeutics during hospital stay in case of enrollment.

### Intervention

After endotracheal intubation we will set the inspired oxygen fraction according to randomization. Patients randomly assigned to the 80% oxygen group will receive an inspired oxygen fraction of 0.8 after intubation; those randomly assigned to the 30% oxygen will receive an inspired oxygen fraction of 0.3 in air. Patients will be blinded to group allocation.

We will use 80% versus 30% oxygen concentration to receive the maximum difference between oxygen concentrations without the risk of causing damage, e.g., increased formation of atelectasis [19]. Moreover, almost all studies investigating the effect of supplemental oxygen on, for example, postoperative wound infection used 80% versus 30% inspired oxygen concentration during surgery [5, 6, 20].

Maintaining a minimum SpO_2_ of at least 93% will be attempted. In the case of SpO_2_ < 93% the following interventions to improve oxygenation will be performed: (1) positive end-expiratory pressure (PEEP) will be increased incrementally by 2 cmH_2_O up to maximally 12 cmH_2_O; (2) recruitment maneuver: the inspiratory pressure will be set at 55 cmH_2_O. The PEEP will be set at 12 cmH_2_O and respiratory rate to six breaths/min or higher. Inspiratory pressure will be set to receive a tidal volume of 6 mL/kg ideal BW. The inspiratory ratio will be set (I:E) to 1:1. Inspiratory pressure will be increased until a plateau pressure reaches 40–50 cmH_2_O. After allowing three breaths while maintaining a plateau pressure of 40–50 cmH_2_O, the respiratory rate and inspiratory pressure will be set back to pre-recruitment parameters and (3) if both interventions are not sufficient to increase SpO_2_, then the inspiratory oxygen fraction will be elevated by 0.1 until 93% SpO_2_ is reached.

### Measurements

#### Baseline information

We will record demographic and morphometric data including age, sex, height, weight, American Society of Anesthesiologists (ASA) physical status and medical history (cardiovascular, pulmonary, neurological, presence of diabetes type 1 and type 2, insulin use and alcohol and tobacco use). We will record the home medication (beta-blockers, ACE inhibitors/angiotensin-1 blockers, diuretics, statins, thienopyridines, anticoagulants, insulin or orally administered antidiabetic drugs, alpha-2 agonists/antagonists). We will perform blood samples to measure baseline values in all patients including NT-proBNP, TnT (high-sensitivity, fifth-generation TnT), hemoglobin, inflammatory parameters, thrombocytes, coagulation parameters, electrolytes, creatinine, albumin, liver tests and copeptin. Blood samples for NT-proBNP and oxidation-reduction will be drawn five times (preoperatively, within 2 h after surgery, on the first and third PODs and within 3 days of discharge). Blood samples for TnT and vWF will be drawn four times (preoperatively, within 2 h after surgery, on the first and third PODs).

All laboratory value will be analyzed at the Department of Laboratory Medicine at the Medical University of Vienna.

#### Perioperative data

Intraoperative data, such as end-tidal sevoflurane concentration, anesthetic agents used, medication, fluid balance, amount and start of vasopressor administration, temperature, hemodynamic and respiratory parameters (cardiac output, stroke volume and corrected flow time (FTc), FiO_2_ and end-tidal CO_2_ will be extracted from the electronic anesthesia information management system. Blood-gas analysis (oxygen partial pressure (pO_2_), CO_2_, hemoglobin, pH, base excess), will be performed hourly during surgery and also recorded.

#### Follow-up

Patients will be followed up for the primary and secondary outcome within 2 h after surgery, on POD 1, POD 3 and within 3 days before discharge. Laboratory measurements for outcome assessments are shown in Table 1. We will further perform a telephone follow-up on the 30th day after surgery to evaluate cardiac events including acute heart failure, myocardial infarction and new onset of atrial fibrillation (Table 2). All follow-up measurements will be performed by an investigator who will be unaware of the randomized allocation. Acute heart failure will be defined as cardiac failure needing medical intervention including therapy with inotropes and extracorporeal membrane oxygenation. Myocardial infarction will be diagnosed according the fourth definition of myocardial infarction [21]. Atrial fibrillation will be diagnosed according to the management guidelines for atrial fibrillation [22].

**Table 2:** Schedule of study events

Timepoints		Study period						
	Enrollment	Intervention			Postoperative period			Close out
	Preoperative visit	Before anesthesia	During surgery	End of surgery for 2 h after surgery	POD 1	POD 3	Hospital discharge	POD telephone follow-up
***Enrollment***								
Eligibility screening	X							
Informed consent	X							
Patient history	X							
Allocation		X						
***Intervention***								
80% inspired oxygen			X					
30% inspired oxygen			X					
Anesthesia/surgery variables			X					
80% via face mask				X				
30% via face mask				X				
Adverse events			X	X				
***Assessments***								
Outcomes (see Table 1)					X	X	X	
Adverse events					X	X	X	X

Key: *POD* postoperative day

#### Data analysis

Randomized groups will be compared for balance in patient characteristics, demographic data, type of surgery, duration of surgery and anesthesia and preoperative baseline laboratory values (including: NT-proBNP, TnT, hemoglobin, hematocrit, leukocytes, thrombocytes, prothrombin time, activated prothrombin time, sodium, potassium, calcium, magnesium, creatinine, albumin, transaminases, C-reactive protein). The primary analysis will be intention to treat so that all randomized patients will be included in all analyses.

The primary outcome, the effect of 80% versus 30% oxygen on postoperative maxNT-proBNP will be compared using a two-sample, two-tailed Mann-Whitney *U* test. If a patient dies before NT-proBNP on POD 3 is measured, the patient’s maxNT-proBNP will be set to the maximum maxNT-proBNP values over all patients. Multiple imputation will be used for missing postoperative measurements.

Our secondary outcomes including Myocardial Injury after Noncardiac Surgery (MINS) (defined as absolute change of high-sensitive TnT of 5 ng/L between 20 to < 65 ng/L) [23], surgical stress, redox stress and perioperative hemodynamic (fluid and vasopressor requirements) will be analyzed using a two-sample, two-tailed Mann-Whitney *U* test. The exploratory outcomes including cardiac failure, myocardial infarction, acute heart failure requiring intervention, new onset of cardiac arrhythmias and unplanned ICU admission will be analyzed using Kaplan-Meier curves assessed with a log-rank test (Table 1).

If hospital discharge occurs before the third POD, the maximum value of the available data will be used for outcome measure.

#### Sample size consideration

A previous study showed a nearly 40% reduction of BNP levels in patients with congestive heart failure receiving nocturnal oxygen therapy [12]. Based on our own data of a previous trial (unpublished data) we observed a fourfold increase of BNP (preoperative 129.05 ng/L ± 160.13 to up to 482.49 ng/L ± 538.78) in the postoperative period in relatively healthy patients undergoing moderate- to high-risk open abdominal surgery. We calculated a needed sample size of 254 patients (127 patients per group) to show a 40% reduction of NT-proBNP in patients receiving 80% versus 30% oxygen with a power of 80% at a 0.05 significance level. We will thus include 260 patients (130 patients per group) to compensate for potential drop outs.

### Assignment to intervention

Given an average estimated recruitment rate of 10 patients per month, we anticipate a recruitment period of 25 months. A trained study coordinator will evaluate eligibility and obtain informed consent. All patients will be recruited using the operation schedule the day before surgery. After meeting the eligibility criteria, the inclusion criteria will be checked. Two copies of the signed consent form will be made. One copy remains in the patient chart, and the second one will be given to the patient. On the consent form, participants will be asked if they agree to the use of their data should they choose to withdraw from the trial. Participants will also be asked for permission for the research team to share relevant data with people from the Universities taking part in the research or from regulatory authorities, where relevant. This trial does not involve collecting biological specimens for storage.

On the day of surgery the coordinator will enroll the participants via accessing the web-based system. We will use permutated block randomization using a web-based randomization program (Randomizer, Medical University of Graz, Graz, Austria). Each block has a size of 6 numbers, randomly ordered to the two treatment assignments, of which all investigators were unaware. The web-based randomization program was set up by an investigator not involved in data acquisition. Intraoperative investigators and clinicians will be not blinded to the allocated treatment group. However, an observer strictly blinded to the group assignment will evaluate complications during hospitalization. Subsequently, a blinded investigator will call all patients 30 days after surgery and to evaluate outcomes.

### Data collection, management and analysis

Data will be recorded in electronic case report forms (eCRFs) and reviewed by the clinical research associate (CRA) during monitoring visits. The CRA will verify recorded data in the electronic data

capture (EDC) system with the stored source documents. All corrections or changes made to any study data will be appropriately tracked in an audit trial in the EDC system An eCRF will be considered completed when all missing, incorrect, and/or inconsistent data have been accounted for.

For data management we will use Clincase software (Version 2.6.0.34, Quadratek, Berlin, Germany).

All records of subjects, source documents, monitoring visit logs and CRFs will be kept locked in the appropriate study files in our site.

#### Monitoring

The designated monitor will contact and visit the investigator on a regularly basis and will be allowed to have access to all source documents, which are needed to verify the entries in the CRF and eCRF, respectively, and other protocol-related documents. It will be the responsibility of the monitor to inspect the CRFs and eCRFs, respectively, at regular intervals according to the monitoring plan throughout the study, to verify the adherence to the protocol and the completeness, consistency and accuracy of the data being recorded. The monitoring will be performed by Karl Schebesta, MD, Department of Anaesthesia, Intensive Care Medicine and Pain Medicine.

### Access to data

Any data required to support the protocol can be supplied on request.

### Ethics and dissemination

#### Data Safety Monitoring Board

The Data Safety Monitoring Board (DSMB) will be formed by Oliver Kimberger, MD, Department of Anaesthesia, Intensive Care Medicine and Pain Medicine, Medical University of Vienna, and Anton Stift, MD, Department of Surgery, Medical University of Vienna. The DSMB will evaluate serious adverse events (SAEs) from the trial after 100 enrolled patients. This committee, along with the Local Ethics Committee will have the exclusive authority to stop the study either because the hypothesis has been confirmed or denied, or because the adverse events are overwhelming. Any morbidity potentially related to the study protocol will be reported to the Ethics Committee.

When the data of 100 patients become available the DSMB will evaluate possible overwhelming harmful effects between both study groups and discuss whether to stop the trial early to prevent harm. The DSMB will monitor for an adverse impact of oxygen on SAEs.

The Trial Steering Group and the independent Data Monitoring and Ethics Committee will meet to review conduct throughout the trial period.

#### Adjudication of the trial outcomes

The outcome adjudicator will be blinded to treatment allocation and will adjudicate the following outcomes: death, myocardial infarction, acute heart failure, cardiac arrhythmias and unplanned ICU admission. We will use the decisions of the outcome adjudicator for all statistical analysis of these events. Barbara Kabon, MD (Department of Anaesthesia, Intensive Care Medicine and Pain Medicine) will chair the Adjudication Committee.

#### Other management at the discretion of the attending physician

The implementation of 80% versus 30% inspired oxygen concentration will not require alteration to usual care pathways (including use of any medication) and these will continue for both trial arms. Furthermore, there will be no anticipated harm and compensation for trial participation. There will be no restriction in the postoperative care regarding medications and therapies. All patients will be treated after completion of the study according to standard clinical care.

All aspects of the patient’s management are at the discretion of the attending physician. This includes all decisions of the intraoperatively used oxygen concentration.

Blinded research personnel will follow patients throughout their time in hospital evaluating the patients and reviewing their medical records ensuring that trial orders are followed and noting any outcomes. If patients indicate that they have experienced any of the outcome measures, the study personnel will obtain the appropriate documentation.

### Reporting of serious adverse events (SAE)

Serious adverse events are those which are fatal, life-threatening or fulfill the definition of being clinically important. Efficacy or safety outcomes will not be considered as SAEs, except if, because of the course of severity or any other feature of such events, the investigator, according to their best medical judgment, considers these events as exceptional in this medical condition. All events considered as part of the primary, secondary, or safety events should be reported on the appropriate page(s) in the CRF but not as an SAE, unless considered exceptional in this medical condition. In this trial, the following events (pulmonary failure, cardiac failure, myocardial infarction, acute heart failure, new onset of cardiac arrhythmias, unplanned ICU admission) are considered related to the underlying cardiovascular disease and are not considered as a SAE. These events will not be considered unexpected unless their course, severity or other specific features are such that the investigator, according to their best medical judgment, considers these events as exceptional in the context of the patient. Only unexpected and not previously described SAEs that are believed with a reasonable level of certainty to be associated with the trial medication need to be reported immediately (i.e., within 24 h of knowledge of the event) to the sponsor of the trial, the Medical University of Vienna. For such events, research personal will complete the SAE CRF immediately. Regulatory authorities will be informed in a timely manner according to the applicable regulations.

## Discussion

This study is designed to evaluate the effect of perioperative supplemental oxygen administration on postoperative NT-proBNP concentrations in patients with increased cardiovascular risk factors undergoing moderate- to high-risk noncardiac surgery. The physiological background of supplemental oxygen is to improve myocardial tissue oxygenation and simultaneously reduce myocardial oxygen consumption mediated by a reduction of the heart rate and cardiac output [11]. A recently published subanalysis of a large clinical trial showed a 30% reduction in the composite of myocardial injury, in hospital cardiac arrest and in mortality in patients undergoing colorectal surgery assigned to the 80% oxygen group [24]. Furthermore, elevated BNP and/or TnT concentrations in the immediate postoperative period are predictive of long-term outcome [16]. Interestingly, a subanalysis of a large outcome trial [25] showed that NT-proBNP and TnT are significantly increased in relatively healthy patients (unpublished data). It seems likely that the stress exposure due to surgery and anesthesia might restrain myocardial contractility, which will be reflected by the elevated NT-proBNP values. Despite the controversy regarding the harmful effects of supplemental oxygen during surgery there is evidence that oxygen might beneficially affect the cardiovascular system. In patients with central sleep apnea, for example, oxygen therapy attenuated the progression of chronic heart failure, which was assessed using BNP measurement [12]. Data of postoperative NT-proBNP concentrations, especially in patients with increased cardiovascular risk factors, are still lacking and are, therefore, of major interest. Thus, we are testing a possible beneficial effect of supplemental oxygen on perioperative myocardial function, which will be obtained by consecutive postoperative NT-proBNP measurements.

Since the effect of hyperoxia on oxidative stress is based mostly on weak studies with small sample sizes we also decided to evaluate the effect of supplemental oxygen on oxidation-reduction potential [26–28]. Measurements of sORP and cORP include all known and unknown oxidants; hence, the determination of sORP and cORP should provide a comprehensive overview of the actual system of oxidative stress.

We wish further to evaluate the immune-modulating effect of supplemental oxygen on endothelial-derived factors [29, 30]. Increased inflammation mediated by hyperoxia might result in elevated vWF-antigen concentrations reflecting endothelial damage.

Previous studies in healthy volunteers indicated a dose-dependent peripheral arterial vasoconstriction, consequently increasing systemic vascular resistance [31]. Thus, we hypothesize that intraoperative supplemental oxygen administration leads to vasoconstriction, which might reduce the required amount of fluid to maintain hemodynamic stability.

### Summary

We plan a randomized clinical trial in patients with increased cardiovascular risk factors undergoing moderate- to high-risk noncardiac surgery to compare the effects of intraoperative 80% inspired oxygen versus 30% inspired oxygen on postoperative maximum NT-proBNP concentrations. Since elevated postoperative NT-proBNP is a strong predictor for postoperative cardiac complications, patients may benefit if the postoperative NT-proBNP release can be reduced by the simple administration of supplemental oxygen.

### Trial status

Actual protocol version 1.4; 19 July 2017. Recruitment started 1 December 2017 and was completed in December 2019.

## Supplementary information

**Additional file 1.** Standard Protocol Items: Recommendations for Interventional Trials (SPIRIT) 2013 Checklist: recommended items to address in a clinical trial protocol and related documents.

## Data Availability

The datasets analyzed during the current study are available from the corresponding author on reasonable request.

## Abbreviations

BNP: Brain natriuretic peptide
BW: Body weight
cORP: Oxidation-reduction potential capacity
DSMB: Data Safety Monitoring Board
FTc: Corrected flow time
MAC: Minimum alveolar concentration
MINS: Myocardial Injury after Noncardiac Surgery
NT-proBNP: *N*-terminal pro-B-type natriuretic peptide
PEEP: Positive end-expiratory pressure
pO_2_: Oxygen partial pressure
POD: Postoperative day
SAE: Serious adverse event
sORP: Static oxidation-reduction potential
TnT: Troponin T
vWF: von Willebrand factor
